# Isolation, Phylogenetic Analysis and Anti-infective Activity Screening of Marine Sponge-Associated Actinomycetes

**DOI:** 10.3390/md8030399

**Published:** 2010-02-26

**Authors:** Usama Ramadan Abdelmohsen, Sheila M. Pimentel-Elardo, Amro Hanora, Mona Radwan, Soad H. Abou-El-Ela, Safwat Ahmed, Ute Hentschel

**Affiliations:** 1 Julius-von-Sachs-Institute for Biological Sciences, University of Würzburg, Julius-von-Sachs-Platz 3, 97082 Würzburg, Germany; E-Mail: sheila-marie.elardo@uni-wuerzburg.de (S.M.P.-E.); Usama.Ramadan@biozentrum.uni-wuerzburg.de (U.R.A.); 2 Research Center for Infectious Diseases, Josef-Schneider-Straße 2, 97080 Würzburg, Germany; 3 Department of Pharmacognosy, Faculty of Pharmacy, Minia University, Minia, Egypt; 4 Department of Microbiology, Faculty of Pharmacy, Suez Canal University, Ismailia, Egypt; E-Mail: ahanora@yahoo.com; 5 Department of Biochemistry, Faculty of Pharmacy, Suez Canal University, Ismailia, Egypt; E-Mails: monasmaa@yahoo.com (M.R.); s_abouelela@yahoo.com (S.H.A.-E.-E.); 6 Department of Pharmacognosy, Faculty of Pharmacy, Suez Canal University, Ismailia, Egypt; E-Mail: safwat_aa@yahoo.com

**Keywords:** actinomycetes, marine sponges, anti-infective, anti-parasitic, phylogenetic analysis

## Abstract

Terrestrial actinomycetes are noteworthy producers of a multitude of antibiotics, however the marine representatives are much less studied in this regard. In this study, 90 actinomycetes were isolated from 11 different species of marine sponges that had been collected from offshore Ras Mohamed (Egypt) and from Rovinj (Croatia). Phylogenetic characterization of the isolates based on 16S rRNA gene sequencing supported their assignment to 18 different actinomycete genera representing seven different suborders. Fourteen putatively novel species were identified based on sequence similarity values below 98.2% to other strains in the NCBI database. A putative new genus related to *Rubrobacter* was isolated on M1 agar that had been amended with sponge extract, thus highlighting the need for innovative cultivation protocols. Testing for anti-infective activities was performed against clinically relevant, Gram-positive (*Enterococcus faecalis*, *Staphylococcus aureus*) and Gram-negative (*Escherichia coli*, *Pseudomonas aeruginosa*) bacteria, fungi (*Candida albican*s) and human parasites (*Leishmania major*, *Trypanosoma brucei*). Bioactivities against these pathogens were documented for 10 actinomycete isolates. These results show a high diversity of actinomycetes associated with marine sponges as well as highlight their potential to produce anti-infective agents.

## 1. Introduction

Infectious disease is the number one cause of death in tropical countries accounting for approximately half of all fatalities. In addition, infectious disease mortality rates are also increasing in developed countries [1]. Emerging and re-emerging infections are thought to be driven largely by socio-economic, environmental and ecological factors [2–4]. Jones *et al*. reported the emergence of 335 infectious diseases between 1940 and 2004 in the global human population [5]. These negative health trends call for a renewed interest in infectious disease as well as effective strategies for treatment and prevention. With respect to the development of new antimicrobials, the marine environment holds great promise for the discovery of novel bioactive compounds.

Marine sponges (phylum Porifera) are among the most ancient multicellular animals (metazoans). These sessile, filter feeding animals are a rich source of novel biologically active metabolites and offer great potential for drug discovery and, in the long term, for treatment of cancer and infectious diseases [6]. Sponges are also known to have intimate contact with various types of microorganisms such as viruses, bacteria, archaea, fungi, protozoa and single-celled algae, and the nature of the sponge-microbe interaction is manifold [7,8]. In general terms, microorganisms serve as food particles, which are retained from seawater in the choanocyte chambers, translocated into the mesohyl interior and digested by phagocytosis. Many sponges contain furthermore symbiotic microbial consortia within their mesohyl matrix that may amount up to nearly half of their biomass. The implementation of the 16S rRNA gene as a phylogenetic marker has, over the last decade, provided unprecedented insights into the microbiology of sponges [7,8]. As many as 18 different prokaryotic phyla and one candidate phylum were so far discovered from sponges, the vast majority of which remains unculturable to this date. Finally, sponges can be overgrown by microbial biofilms and can even succumb to infections much like has been reported for corals and other invertebrates [9].

Members of the phylum *Actinobacteria* and specifically the order *Actinomycetales* have been identified as abundant members of sponge-associated microbial communities [10–15]. Their existence in the marine environment has been further shown in marine sediments as well as in the deepest ocean trenches [16–21]. Actinomycetes are of considerable interest owing to their ability to produce new chemical entities with diverse pharmacological activities. Marine actinomycetes in particular have yielded numerous novel secondary metabolites [22]. New actinomycete taxa of marine origin have also been recovered as best exemplified by *Salinispora*, the first marine obligate actinomycete isolated from ocean sediments [23] as well as from a sponge [24]. Our aim is to isolate and culture actinomycetes from marine sponges and to characterize their potential to produce bioactive compounds, specifically those which inhibit the growth of human pathogens and parasites.

## 2. Results and Discussion

### 2.1. Diversity of Sponge-Associated Actinomycetes

**Figure 1:**
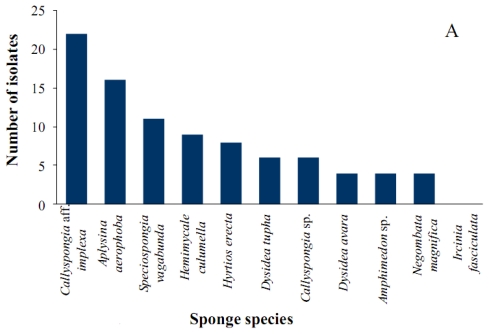

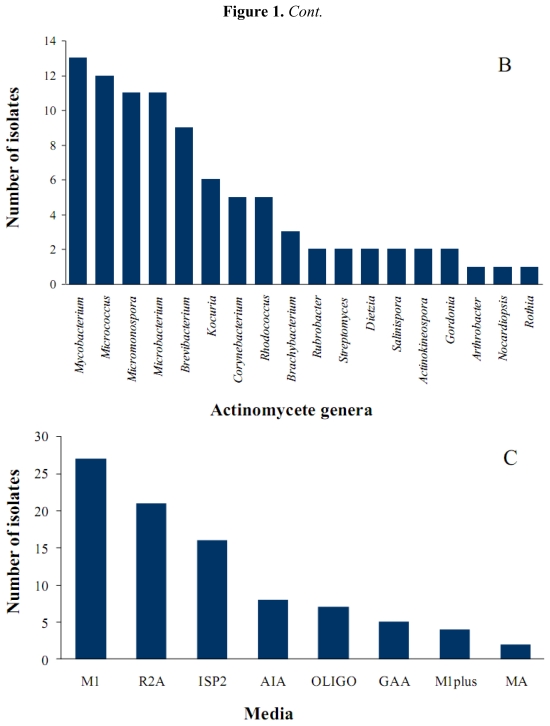
Number of actinomycete isolates (A) per sponge species, (B) per actinomycete genera and (C) per cultivation media.

Homogenates from 11 taxonomically different marine sponges collected from Ras Mohamed, Egypt and Rovinj, Croatia were plated on a range of selective media for the isolation of actinomycetes. *Callyspongia* aff. *implexa* yielded the highest number of isolates (22), followed by *Aplysina aerophoba* (16), *Spheciospongia vagabunda* (11), *Hemimycale culumella* (9), *Hyrtios erecta* (8), *Dysidea tupha* (6), *Callyspongia* sp. (6), *Dysidea avara* (4), *Amphimedon* sp. (4), *Negombata magnifica* (4), while no actinomycete strains were cultivated from *Ircinia fasciculata* (Figure 1A). A comparison of the nearly complete 16S rRNA gene sequences of 52 out of 90 putative actinobacterial strains isolated from these sponges against sequences in the NCBI GenBank database revealed the phylogenetic affiliations to 18 different genera representing 14 families and seven suborders (Supplementary Table 1). The highest number of isolates were found to be affiliated with the genus *Mycobacterium* (13), followed by *Micrococcus* (12), *Micromonospora* (11), *Microbacterium* (11), *Brevibacterium* (9), *Kocuria* (6), *Corynebacterium* (5), *Rhodococcus* (5), *Brachybacterium* (3), *Rubrobacter* (2), *Streptomyces* (2), *Dietzia* (2), *Salinispora* (2), *Actinokineospora* (2), *Gordonia* (2), *Arthrobacter* (1), *Nocardiopsis* (1) and *Rothia* (1) (Figure 1B). It has been reported that the genera belonging to *Micromonospora*, *Rhodococcus* and *Streptomyces* are the dominant actinobacterial genera in marine environments [25]. Representatives of all three taxa were isolated from the marine sponges studied here although other genera such as *Mycobacterium*, *Micrococcus, Microbacterium* were also represented by high numbers of isolates.

Given that less than 1% of bacteria associated with sponges can be cultured, the use of appropriate isolation media is critical for improving the recovery of actinobacteria [14]. The isolation media of this study were chosen to select for actinobacteria. M1 exhibited the highest recovery producing 27 isolates and MA recovered only two isolates (Figure 1C). In terms of diversity, M1 also produced isolates belonging to the highest number of genera (13), followed by ISP medium 2 (11 genera), R2A (eight genera), AIA (six genera), M1 plus (five genera), OLIGO (four genera), GAA (two genera), and the lowest diversity was observed with MA which recovered only one genus. This variation likely reflects the effect of media composition, which is consistent with previous observations [14,25]. Addition of aqueous sponge extract to M1 medium resulted in the isolation of a putatively new genus with *Rubrobacter* as its closest relative. This shows that variation from standard protocols is a worthwhile procedure. It was reported that media without added sponge extract produced the largest number of different colony morphotypes and the addition of sponge extract resulted in an increase in the number of novel cultivated morphotypes [26].

**Figure 2:**
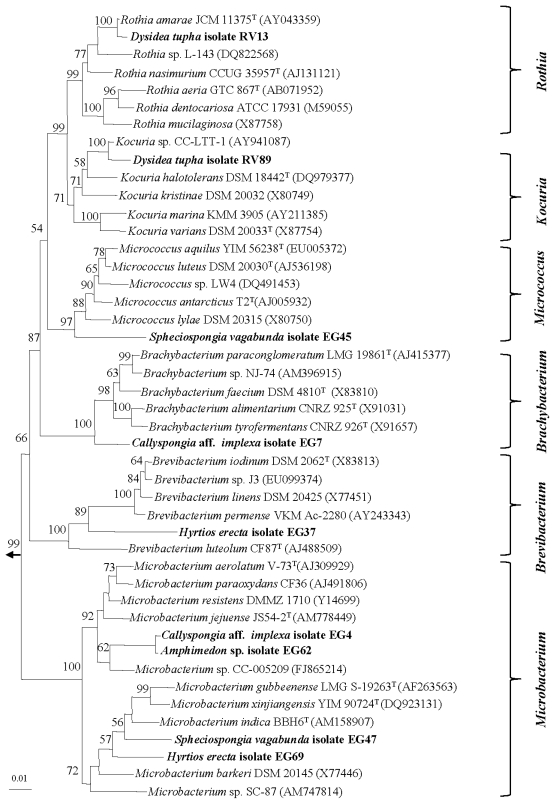
Neighbor-joining tree of the strains and representative species of the suborder *Micrococcineae* based on nearly complete 16S rRNA gene sequences. Numbers at the nodes indicate the levels of bootstrap support based on 1000 resampled data sets. Only values greater than 50% are shown. The arrow points to the outgroup consisting of five species belonging to *Enterobacteriaceae.* The scale bar indicates 0.01 substitution per nucleotide position.

**Figure 3:**
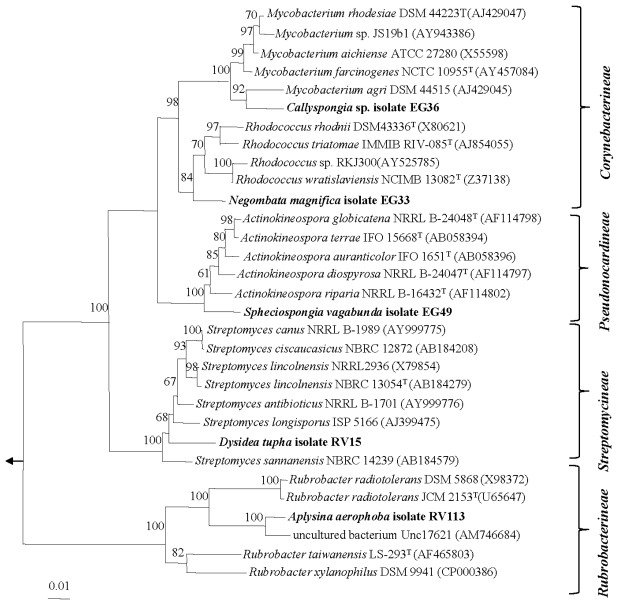
Neighbor-joining tree of the strains and representative species of the suborders *Corynebacterineae*, *Pseudonocardineae*, *Streptomycineae* and *Rubrobacterineae* based on nearly complete 16S rRNA gene sequences. Numbers at the nodes indicate the levels of bootstrap support based on 1000 resampled data sets. Only values greater than 50% are shown. The arrow points to the outgroup consisting of five species belonging to Enterobacteriaceae. The scale bar indicates 0.01 substitution per nucleotide position.

Interestingly, fourteen strains exhibited only 92.9–98.2% sequence similarities with validly described species. The low sequence similarities suggest that these strains belong to novel actinomycete taxa which are further supported by phylogenetic analysis [27] (Figures 2 and 3). These putatively novel isolates were affiliated to the genera *Rothia*, *Kocuria*, *Micrococcus*, *Brachybacterium*, *Brevibacterium*, *Microbacterium* (suborder *Micrococcineae*) (Figure 2). The isolates EG4 and EG62 from *Callyspongia* sp. and *Amphimedon* sp., respectively, formed a separate cluster from close relatives of the genus *Microbacterium*. The high sequence similarity (99.8%) of the isolates with only three nucleotide differences suggests that they are the same strain although they were isolated from different sponge species. The other putatively novel isolates were affiliated with the following genera: *Mycobacterium* and *Rhodococcus* (suborder *Corynebacterineae)*, *Actinokineospora* (suborder *Pseudonocardineae*), *Streptomyces* (suborder *Streptomycineae*) and *Rubrobacter* (suborder *Rubrobacterineae*) (Figure 3). Interestingly, isolate RV113 from *Aplysina aerophoba* exhibited 92.9% sequence similarities to *Rubrobacter radiotolerans* as well as to several uncultured clones. Phylogenetic analysis revealed that the isolate RV113 forms a distinct clade and the low sequence similarity values further suggest that RV113 possibly belongs to a novel genus with *Rubrobacter* as its closest relative. Phenotypic and genotypic characterization will be pursued to validate the taxonomic position of this strain as well as the remainder of putatively novel actinomycete isolates cultivated in this study.

Several studies have previously reported on the isolation and diversity of actinobacteria from marine sponges. Montalvo *et al.* [13] showed that actinobacteria are major components of the microbial communities of *Xestospongia muta* and *X. testudinaria*. Zhang *et al*. [15] isolated 106 actinomycete strains representing seven genera from the sponge *Hymeniacidon perleve*. Additionally, 181 culturable actinomycetes affiliated with three genera were recovered from five sponges offshore China [25]. The microbial diversity of two Red Sea sponges, *Hyrtios erectus* and *Amphimedon* sp., were recently inspected using cultivation and cultivation-independent analyses. Focused cultivation on actinobacteria yielded 35 actinomycetes represented by four genera. [28]. Specifically, strains belonging to *Actinoalloteichus*, *Brachybacterium*, *Brevibacterium, Curtobacterium*, *Gordonia*, *Kocuria*, *Micrococcus*, *Micromonospora*, *Nocardiopsis*, *Rhodococcus*, *Salinispora* and *Streptomyces* were previously isolated from marine sponges and may therefore be common components thereof [13,15,28–30]. However, none of the strains isolated in our study showed sequence similarities with those previously reported from sponges, thus rendering the existence of sponge-specific actinomycete clades in the sense of Hentschel *et al*. [10] unlikely.

### 2.2. Bioactivity Testing

A major goal of our study is to establish new sources of antibiotics and other bioactive compounds. In order to prevent reisolation of already known compounds, which is a common problem in natural products discovery, the 14 putatively novel strains plus six additional ones based on their moderate relatedness to known metabolite producers were selected for bioactivity screening. The discovery rate of novel compounds is indeed much higher in taxonomically novel than in known strains [31]. The antimicrobial activity was assessed by the disc diffusion assay while the anti-parasitic activity was measured in multi-well plates and is represented as the percentage of growth inhibition in comparison to a reference compound. Of the twenty strains tested, ten produced antimicrobially active metabolites inhibiting at least one of the test pathogens. Five isolates were capable of inhibiting the growth of Gram-positives only, one isolate was active against *C. albicans* only and one isolate showed activity against both groups of pathogens (Table 1). The inhibition was greatest with the *Streptomyces* sp. RV15 against *Staphylococcus aureus*. Gram-negative bacteria were not susceptible to our isolates. In terms of the anti-parasitic activity, four isolates exhibited activity against *T. brucei* and two isolates showed activity against *L. major* (Table 2). The novel *Actinokineospora* sp. EG 49 showed highest antitrypanosomal potential at 48% growth inhibition. Anti-parasitic activities from marine-derived actinomycetes are reported only sporadically. For example, a new thiolactone antibiotic, thiolactomycin, from *Nocardia* strain No. 2–200 showed potent antimalarial and antitrypanosomal activities [32]. Additionally, the macrolide antibiotic amphotericin B produced by *Streptomyces nodosus* was used for treatment of leishmaniasis [33].

Because crude extracts were tested for bioactivities, the chemical nature of the bioactive compound is currently unknown. Nevertheless, some genera, to which the novel isolates belong to, are well known and prolific metabolite producers. For example, the genus *Streptomyces* accounts for about 80% of all natural products recovered from actinomycetes to date [34]. As one example, *Streptomyces sannanensis* produces new aminoglycoside antibiotics sannamycins A, B and C with potent activities against Gram-positive bacteria [35]. First organic chemistry analyses of the novel isolate RV15 points to entirely novel cyclic peptides which are currently being characterized further. *Rhodococcus* species, of which one putatively novel and bioactive strain was isolated in this study, produce a number of commercially interesting and potentially useful products including various types of steroids and peptides [36]. For example, two antimycobacterial cyclic peptides, lariatins A and B, were isolated from the culture broth of *Rhodococcus* sp. K01-B0171 [37]. The genus *Microbacterium*, of which two putatively new species were isolated in this study, produces different glycolipids. For example, five anti-tumor glycoglycerolipids were obtained from *Microbacterium* sp., which was isolated from the sponge *Halichondria panicea* [38]. Antimicrobial activities have, to our knowledge, not been reported for the genera *Actinokineospora*, *Kocuria* and *Rubrobacter* and the identification of metabolites produced by the putative new species of these genera is currently underway.

In conclusion, considerable actinobacterial diversity was recovered from various marine sponges. Altogether 90 actinomycete isolates were affiliated with 18 different genera including 14 putatively novel species. One possibly new genus related to *Rubrobacter* was isolated on M1 agar amended with sponge extract, thus highlighting the need for innovative cultivation media. Antibacterial, antifungal, antitrypanosomal and antileishmanial activities were reported for 10 of the isolates. Marine sponges represent therefore a still largely untapped resource for novel actinomycete diversity as well as for new secondary metabolites of therapeutic value.

## 3. Experimental

### 3.1. Sponge Collection

The first group of sponges (*Aplysina aerophoba*, *Dysidea avara*, *D. tupha*, *Hemimycale culumella*, *Ircinia fasciculata*) was collected by SCUBA diving at depths of 3–20 m in the Mediterranean Sea (Rovinj, Croatia, (GPS: 27°47.655 N; 34°12.904 W) in August 2008. Taxonomic identification was performed by W.E.G. Müller and I. Müller (University of Mainz, Germany). The second group (*Amphimedon* sp., *Callyspongia* sp., *C.* aff. *implexa*, *Hyrtios erecta*, *Negombata magnifica, Spheciospongia vagabunda*) was collected at a depth of 10 m in the Red Sea (Ras Mohamed, Sinai, Egypt; (GPS: 27°47.655 N; 34°12.904 W) in August 2006. *Amphimedon* sp. and *Hyrtios erecta* were identified by M. Kelly (National Institute of Water and Atmospheric Research (NIWA) Auckland, New Zealand) and the remaining sponges by R.W.M. van Soest (University of Amsterdam, Netherlands). Sponges were transferred to plastic bags containing seawater and transported to the laboratory. Sponge specimens were rinsed in sterile seawater, cut into pieces of ca. 1 cm^3^, and then thoroughly homogenized in a sterile mortar with 10 volumes of sterile seawater. The supernatant was diluted in ten-fold series (10^−1^, 10^−2^, 10^−3^) and subsequently plated out on agar plates.

### 3.2. Actinomycete Isolation

Eight different media [M1 [19], ISP medium 2 [39], Oligotrophic medium (OLIGO) [40], M1 plus [14], Actinomycete Isolation Agar (AIA) [41], Marine Agar (MA) [42], Glycerol Asparagine Agar (GAA) [41] and R2A Agar [43]] were used for the isolation of actinobacteria. All media were supplemented with 0.2 μm pore size filtered cycloheximide (100 μg/mL), nystatin (25 μg/mL) and nalidixic acid (25 μg/mL) to facilitate the isolation of slow-growing actinobacteria. Cycloheximide and nystatin inhibit fungal growth, while nalidixic acid inhibits many fast-growing Gram-negative bacteria [14]. All media contained Difco Bacto agar (18 g/L) and were prepared in 1 L artificial sea water (NaCl 234.7 g, MgCl_2_.6 H_2_O 106.4 g, Na_2_SO_4_ 39.2 g, CaCl_2_ 11.0 g, NaHCO_3_ 1.92 g, KCl 6.64 g, KBr 0.96 g, H_3_BO_3_ 0.26 g, SrCl_2_ 0,24 g, NaF 0.03 g and ddH_2_O to 10.0 L) [44]. To promote the growth of selected sponge-associated actinobacteria, 1% “aqueous sponge extract” was added to the autoclaved medium. Aqueous sponge extract was prepared by grinding 20 g of sponge biomass in a mortar containing 20 mL of sterile seawater followed by centrifugation (5000 rpm, 10 min) and sterilized by filtration through a 0.2 μm pore size filter. The freshly prepared supernatant served as aqueous sponge extract. The inoculated plates were incubated at 30 °C for 6–8 weeks. Distinct colony morphotypes were picked and re-streaked until visually free of contaminants. Isolates were inoculated into liquid media (M1 or the medium on which colonies were initially isolated). The isolates were maintained on plates for short-term storage and long-term strain collections were set up in medium supplemented with 30% glycerol at −80 °C. The isolates from Egypt are abbreviated as “EG” and from Rovinj as “RV”.

### 3.3. Molecular Identification and Phylogenetic Analysis

16S rRNA gene amplification, cloning and sequencing were performed according to Hentschel *et al*. [45] using the universal primers 27F and 1492R [46]. Chimeric sequences were identified by using the Pintail program [47]. The genus-level affiliation of the sequences was validated using the Ribosomal Database Project Classifier [48]. Sequence alignment and phylogenetic analysis were performed using the ARB software package [49]. Tree construction was conducted using neighbour-joining algorithm (Jukes-Cantor correction) with bootstrap values based on 1000 replications. The 16S rRNA gene sequences of the putatively novel isolates were deposited in GenBank under the accession numbers indicated in parentheses: EG4 (GU318354), EG7 (GU318355), EG33 (GU318356), EG36 (GU318357), EG37 (GU318358), EG45 (GU318359), EG47 (GU318360), EG49 (GU318361), EG62 (GU318362), EG69 (GU318363), RV15 (GU318364), RV113 (GU318365), RV13 (GU318366) and RV89 (GU318367).

### 3.4. Extract Preparation and Anti-infective Activity Screening

Fourteen strains selected based on phylogenetic novelty and six selected based on their affiliation to known metabolite-producers were cultured in 100 mL Erlenmeyer flasks containing 50 mL of 5 different production media (M1, ISP2, OLIGO, AIA and R2A) for each isolate. The liquid cultures were grown for 7–14 days depending on their growth rate at 30 °C while shaking at 150 rpm. An equal volume of methanol was added to the liquid cultures for cell lysis and shaking was continued (150 rpm, 1 h at room temperature; Shaker SM 30, E. Bühler). The broth was centrifuged in 50 mL falcon tubes (5000 rpm, 15 min at room temperature; Megafuge 1.0R, Heraeus) and the supernatant was stored at 4 °C.

The *in vitro* antimicrobial activity testing was carried out using the standard disk diffusion assay [50] against pathogenic bacteria (*Staphylococcus aureus* strain 8325, *Enterococcus faecalis* strain JH212, *Escherichia coli* strain 536, *Pseudomonas aeruginosa* strain Nr. 3) and yeast (*Candida albicans* strain S314). Sterile filter disks (6 mm) impregnated with actinomycete extracts were placed on agar plates that had been inoculated with the test pathogen. After 24 h incubation at 37 °C (bacteria) and 30 °C (yeast), the antimicrobial potential was quantitatively assessed as diameter of the inhibition zone (n = 2). Anti-leishmanial activity was tested following the method of Ponte-Sucre *et al.* [51]. Briefly, this involved the incubation of *Leishmania major* promastigotes for 24 h at 26 °C, 5% CO_2_, and 95% humidity in the absence or presence of the extracts. Following the addition of Alamar Blue, the plates were incubated again and the optical densities were determined after 48 h with a Multiskan Ascent enzyme-linked immunosorbent assay (ELISA) reader (Thermo Electron Corporation, Dreieich, Germany). Absorbance in the absence of compounds was set as 100% of growth. Amphotericin B was used as a reference compound and positive control. Each extract was assayed in duplicate from two independent experiments. Anti-trypanosomal activity was tested following the protocols of Huber and Koella [52]. Following cultivation of *Trypanosoma brucei brucei* strain TC 221 in Complete Baltz Medium, a defined number of parasites were exposed in 96-well plate test chambers to the extracts. The plates were then incubated at 37 °C in an atmosphere of 5% CO_2_ for 24 h. After addition of Alamar Blue, the activity of the extracts was measured by light absorption using MR 700 Microplate Reader after 48 h. Absorbance in the absence of compounds was set as 100% of growth. Each extract was assayed in duplicate from two independent experiments.

## Figures and Tables

**Table 1:** Antimicrobial activities of sponge-derived actinomycetes.

Sponge isolate	Inhibition zone diameter (mm)		
	*S. aureus* 8325	*E. faecalis* JH212	*C. albicans* S314
*Actinokineospora* sp. EG49*	0	0	12
*Dietzia* sp. EG67	13	0	0
*Microbacterium* sp. EG69*	10	9	0
*Micromonospora* sp. RV115	12	10	0
*Rhodococcus* sp. EG33*	12	8	0
*Rubrobacter* sp. RV113**	9	0	0
*Streptomyces* sp. RV15*	17	11	13

*: putatively new species;

**: putatively new genus.

**Table 2:** Anti-parasitic activities of sponge-derived actinomycetes.

Sponge isolate	% growth inhibition	
	*L. major*	*T. brucei TC 221*
*Actinokineospora* sp. EG49*	24	48
*Brevibacterium* sp. EG10	0	30
*Gordonia* sp. EG50	36	28
*Kocuria* sp. RV89*	0	19

*: putatively new species.
